# Sex-specific aspects in a population of patients undergoing evaluation for interstitial lung disease with transbronchial cryobiopsy

**DOI:** 10.1038/s41598-025-94575-0

**Published:** 2025-04-05

**Authors:** Janna Reinhard, Markus Polke, Katharina Buschulte, Monika Eichinger, Claus Peter Heußel, Maximilian Güttlein, Julia D. Michels-Zetsche, Mark Oliver Wielpütz, Kathrin Kahnert, Konstantina Kontogianni, Laura V. Klotz, Michael Allgäuer, Felix J. Herth, Ralf Eberhardt, Michael Kreuter, Franziska C. Trudzinski

**Affiliations:** 1 Department of Pneumology and Critical Care Medicine, Center for Interstitial and Rare Lung Diseases, Translational Lung Research Center Heidelberg (TLRC-H), German Center for Lung Research (DZL), Thoraxklinik University of Heidelberg, Heidelberg, Germany; 2 Translational Lung Research Center Heidelberg (TLRC-H), German Center for Lung Research (DZL), Heidelberg, Germany; 3 Department of Diagnostic and Interventional Radiology with Nuclear Medicine, Thoraxklinik, University Medical Center Heidelberg, Heidelberg, Germany; 4 Department of Diagnostic & Interventional Radiology, University Hospital of Heidelberg, Heidelberg, Germany; 5 MediCenterGermering, Germering, Germany; 6 Department of Medicine V, LMU University Hospital, LMU Munich, Comprehensive Pneumology Center, Member of the German Center for Lung Research (DZL), Munich, Germany; 7 Department of Thoracic Surgery, Thoraxklinik at Heidelberg University Hospital, Heidelberg, Germany; 8 Department of Pathology, University of Heidelberg, Heidelberg, Germany; 9 Department of Pneumology and Intensive Care Medicine, Asklepios Klinik Barmbek, Hamburg, Germany; 10 Departments of Pneumology, Mainz Center for Pulmonary Medicine, Mainz University Medical Center and of Pulmonary, Critical Care & Sleep Medicine, Marienhaus Clinic Mainz, Mainz, Germany

**Keywords:** Interstitial lung disease, Idiopathic pulmonary fibrosis, Quantitative CT analysis, Computed tomography, Sex, Gender, Transbronchial lung cryobiopsy, Respiratory tract diseases, Outcomes research

## Abstract

There are well-documented differences in idiopathic pulmonary fibrosis (IPF) between sexes. The sex-specific prevalence of interstitial lung disease (ILD) subtypes in patients who require a full diagnostic work-up, including transbronchial cryobiopsy (TCB), after initial multidisciplinary discussion (MDD) is still unknown. Retrospective analysis of sex dispareties in patients with ILD who received an interdisciplinary indication for lung biopsy and underwent bronchoalveolar lavage, TCB and, if necessary, surgical lung biopsy at our ILD centre in Heidelberg between 11/17 and 12/21. The analysis included clinical parameters, visual assessment of computed tomography (CT), automated histogram analyses of lung density by validated software and final MDD-ILD classifications. A total of 402 patients (248 men, 154 women; mean age 68 ± 12 years) were analysed. Smoking behaviour was similar between the sexes, but women were more exposed to environmental factors, whereas men were more exposed to occupational factors. Women had higher rates of thyroid disease (29.9% vs. 12.5%; p < 0.001) and extrathoracic malignancies (16.2% vs. 9.3%; p = 0.041), but lower rates of coronary heart disease (7.1% vs. 19.8%; p < 0.001), stroke (1.3% vs. 6.5%; p = 0.014) and sleep apnoea (5.8% vs. 17.7%; p < 0.001). There were no sex differences regarding CT lung density. On visual inspection, women were less likely to have reticular opacities (65% vs. 76%; p = 0.017) and features of usual interstitial pneumonia (17% vs. 34%; p < 0.001). Among final diagnoses, hypersensitivity pneumonitis was more common in women (34.4%) compared to men (21.8%; p = 0.007). In contrast, IPF was more common in men (22.6%) than in women (7.1%; p < 0.001), and unclassifiable interstitial lung disease was also more frequent in men (21.8%) compared to women (6.5%; p < 0.001). This study highlights significant sex-based differences in the prevalence and characteristics of ILD requiring comprehensive diagnostic work-up. These findings underscore the importance of considering sex-specific factors in the diagnosis and management of ILD.

## Introduction

The group of interstitial lung diseases (ILDs) comprises a large and very heterogeneous group of entities with different causes and pathophysiological backgrounds. In the case of ILDs with a known aetiology, such as connective tissue diseases (CTDs) and pneumoconiosis, it is known that sex and gender influence the disease, particularly with regard to different exposure and risk factors. Within the affected population, men were more likely to work in industrial and manual occupations and therefore have a higher risk of exposure to silica and asbestos which are associated with ILDs. Women, on the other hand, are much more likely to have autoimmune diseases and corresponding CTD ILDs. However, despite the higher prevalence of rheumatoid arthritis (RA) and systemic sclerosis (SSc) in women, male sex is a risk factor for developing ILDs when having one of these CTDs^1,2^.

There are significant sex differences in the prevalence of idiopathic interstitial pneumonias (IIPs), such as idiopathic pulmonary fibrosis (IPF), idiopathic non-specific interstitial pneumonitis (iNSIP) or smoking-related subtypes of ILD. These are best studied in IPF, a disease that occurs predominantly in older male smokers; approximately 70% of patients enrolled in large prospective cohorts are male^3^. Sex differences in granulomatous ILD are best studied in hypersensitivity pneumonitis (HP) and sarcoidosis. Sarcoidosis is more common in women, beyond which there are also sex differences in the prevalence of extrapulmonary phenotypes, including cardiac and cutaneous sarcoidosis^4^. Although the sex ratio in HP is probably balanced, the occupational exposure to certain aetiological antigens in particular is different in men and women, which can lead to different clinical manifestations and outcomes^5^. In the group of other rarer entities, the sex distribution in lymphangioleiomyomatosis (LAM), which almost exclusively affects women, is a classic example of the influence of sex in ILDs.

The careful categorisation of ILDs requires a thorough anamnestic and clinical examination and comprehensive expertise in this area^6^. In view of the complexity of the underlying diseases and their broad differential diagnosis, the diagnosis is made in a multidisciplinary ILD board (MDD) after demonstration of all relevant clinical and radiological findings. Primary unclassifiable ILDs represent a minority group in multidisciplinary discussions, with the need for further invasive diagnostic procedures, such as transbronchial lung cryobiopsy (TCB) or surgical lung biopsy (SLB)^7^, indicated in less than 30% of cases, as reported in the literature. We chose to focus on this cohort due to the complexity and uncertainty surrounding their diagnosis. The aim of this monocentric retrospective observational study was to investigate sex-specific aspects regarding prevalence and characteristics of different ILD entities in such a cohort of patients with yet undiagnosed ILD with the indication for further invasive work-up. For this purpose, the data of patients presenting to our centre for bronchoscopy with TCB were systematically analysed.

## Methods

### Study design and participants

This is a retrospective analysis of consecutive patients with suspected ILD evaluated at the Thorax Clinic of the University of Heidelberg, a tertiary referral centre for interstitial lung disease. All patients who underwent transbronchial lung cryobiopsy between November 2017 and December 2021 were included. In case of inconclusive histological findings, the patients were offered a surgical lung biopsy by video-assisted thoracoscopic surgery (VATS) for further classification after MDD. The study was approved by the institutional review board (S-382/2023) and conducted in accordance with the principles of the Declaration of Helsinki. Due to its retrospective design, the requirement for informed consent was waived by the Ethics Committee of the University of Heidelberg.

### Pulmonary function tests

Pulmonary function tests were performed using a Jaeger MasterScreen Body System (CareFusion, Rolle, Switzerland). Lung volumes were assessed by whole-body plethysmography and diffusion capacity for carbon monoxide (DLCO) by the single breath technique. All measurements were performed according to the American Thoracic Society (ATS) and the European Respiratory Society (ERS) guidelines^8–10^, using Global Lung Function Initiative (GLI) equations to express the results as % predicted^11,12^.

### Imaging

A standardised non-contrast thin-section computed tomography (SOMATOM Definition AS, Siemens Healthineers AG) was used for diagnosis if no external imaging of comparable quality was available. The CT images of all patients were presented and evaluated in an interdisciplinary case discussion involving one of three different radiologists, each with several years of experience in chest imaging. Moreover, CT datasets (i40f kernel) were post-processed using the validated in-house software YACTA (version v2.9.4.65) as previously described^13–16^. The segmentation of the airway tree and lung lobes was performed using a fully automated process. The segmentation results were then visually checked for inconsistencies by a reader (J.R.). The houndsfield units (HU) value of the range 10th-90th percentile in 5th percentiles steps was derived from the histogram recording the densities of all lung voxels for each Modified Discrete Cosine Transformation (MDCT). The analyses included the density values of the 40th and 80th percentiles of the MDCT attenuation frequency histogram, as these have been identified in previous studies as parameters of disease extent^15,17^.

### Multidisciplinary board discussion

The multidisciplinary discussion (MDD) team consisted of two experienced pulmonologists, a thoracic radiologist, a thoracic pathologist, and, in the case of suspected systemic disease, a rheumatologist. All cases were discussed at least twice to ensure a thorough evaluation. At the MDD, each patient’s detailed medical history, including exposures and signs of systemic disease, pulmonary function tests, laboratory values, 6-min walk test, echocardiography and high-resolution computed tomography were reviewed according to current national and international guidelines^7,18,19^. Further invasive diagnostic procedures including transbronchial cryobiopsy were also indicated in the multidisciplinary discussions. Finally, all data including histology were discussed again in a second MDD and the final diagnosis was again made according to national and international guidelines^7,18,19^.

### Bronchoscopy

Bronchoscopy and periprocedural preparations were performed according to an institutional standard as previously described^20^. Endoscopists were instructed to collect samples from both the upper and lower lobes of the most affected lung side, utilizing fluoroscopic guidance for precision. They were further advised to obtain samples from the distal part of the lung parenchyma, ensuring a distance of at least 1 cm from the pleura to ensure representation of the peripheral lung areas, as recommended^21^. The commercially available 1.7 or 1.9-mm cryoprobe (ERBE, Solingen, Germany) were used. Two hours after the end of the procedure, a chest x-ray was performed to rule out a pneumothorax.

### Categorisation of ILD

ILDs were categorised into four categories according to the national German ILD guideline^6,22^: Category I: ILDs associated with known causes, Category II: idiopathic forms, Category III: granulomatous ILDs, Category IV: others.

### Statistical analysis

Data in the tables are presented as numbers and percentages or means and standard deviations (SD). Data were analysed with Fisher’s exact test for categorical variables or a two-tailed t test for independent samples in the case of continuous variables. All analyses were performed using SPSS version 25 (IBM Corp., Armonk, NY, USA); p < 0.05 was considered statistically significant.

## Results

### Medical history, clinical and functional characteristics

A total of 402 patient cases, including 145 women and 248 men, were included in the analysies. There were no differences between men and women with regards to age or duration of symptoms before presentation to the centre. Men had a significantly higher body mass index than women (29.3 ± 4.7 vs. 27.8 ± 6.1; p = 0.016). Smoking status and pack-years did not differ between men and women. In the cohort, women reported significantly more environmental (55.8% vs. 41.9%; p = 0.008) and men more occupational (9.3% vs. 1.9%, p = 0.003) exposures, as well as more frequent dust exposures in both areas (19.8% vs. 6.5%, p < 0.001). There were no differences between the sexes in the results of the autoimmune serologies performed. Overall, women had lower values for static and dynamic lung function parameters and diffusion capacity, but there were no differences compared to the corresponding reference values. In a total of 18 patients (4.5%), a surgical lung biopsy was indicated following a transbronchial cryobiopsy (TCB) because the TCB did not yield conclusive results, as determined by the multidisciplinary discussion (MDD). Patient characteristics, stratified by sex, are shown in Table 1.

**Table 1:** Patient characteristics, stratified by sex.

	All N = 402	Females N = 154	Males N = 248	p
Age (years)	67.8 ± 11.8	66.7 ± 13.2	68.6 ± 10.8	0.138
BMI	28.6 ± 5.3	27.8 ± 6.1	29.3 ± 4.7	**0.016**
Symptom onset (month)	19.3 ± 22.2	19.3 ± 23.0	19.3 ± 20.9	0.983
Smoking status (yes)	243 (60.4%)	99 (64.3%)	144 (58.1%)	0.294
Pack years	16.4 ± 22.0	16.7 ± 21.3	16.9 ± 22.4	0.866
Exposition				
Occupational	26 (6.5%)	3 (1.9%)	23 (9.3%)	**0.003**
Environmental	190 (47.3%)	86 (55.8%)	104 (41.9%)	**0.008**
Feather bedding	58 (14.4%)	42 (16.9%)	16 (10.4%)	0.080
Dusts	59 (14.7%)	10 (6.5%)	49 (19.8%)	** < 0.001**
Pets	65 (16.2%)	25 (16.2%)	40 (16.1%)	1.000
Birds	46 (11.4%)	32 (14.9%)	23 (9.3%)	0.106
Serology				
ANA titre ≥ 1:160	120 (30.2%)	47 (30.7%)	73 (29.8%)	0.911
Rheumatoid factor (pos.)	39 (9.8%)	16 (10.5%)	23(9.4%)	0.732
Anti-CCP (pos.)	9 (2.3%)	5 (2%)	4(2.6%)	0.737
Pulmonary function				
FEV1 (L)	2.4 ± 0.7	2.0 ± 0.5	2.7 ± 0.7	** < 0.001**
FEV1 (%predicted)	84.1 ± 20.6	83.1 ± 20.9	84.7 ± 20.4	0.457
FVC (L)	2.9 ± 0.9	2.3 ± 0.6	3.3 ± 0.8	** < 0.001**
FVC (%predicted)	77.1 ± 19.4	77.0 ± 19.8	77.2 ± 19.2	0.925
DLCO (mmol/min/kPa)	4.8 ± 1.6	4.3 ± 1.2	5.2 ± 1.7	** < 0.001**
DLCO (%predicted)	56.6 ± 16.3	57.6 ± 16.2	56.0 ± 16.3	0.323
VATS	18 (4.5%)	4 (2.6%)	14 (5.6%)	0.215

Significant values are in [bold].

The table contains a comparative analysis of Patient characteristics in male and female patients within the study population. Data were analysed with Fisher’s exact test for categorical variables or a two-tailed t test for independent samples in the case of continuous variables; p < 0.05 was considered statistically significant.

FEV1: forced expiratory volume in the first second; FVC: forced Vital capacity; DLCO: diffusing capacity of the lungs for carbon monoxide; VATS: video-assisted thoracic surgery.

### Comorbidities

Several comorbidities were systematically analysed and differences were found between the sexes: Men were significantly more likely to have coronary heart disease (3.6% vs. 0.6%, p < 0.001), sleep apnoea (1.3% vs. 0.4%, p < 0.001) and a history of stroke (6.5% vs. 1.3%, p = 0.014). Women were significantly more likely to have malignancies (16.2% vs. 9.3%, p = 0.041) and thyroid disease (29.9% vs. 12.5%; p < 0.001). The distribution of comorbidities by sex is shown in Table 2.

**Table 2:** Distribution of comorbidities by sex.

	All N = 402	Females N = 154	Males N = 248	p
Peripheral arterial disease	10 (2.5%)	1 (0.6%)	9 (3.6%)	0.097
Coronary heart disease	60 (14.9%)	11 (7.1%)	49 (19.8%)	** < 0.001**
Arterial hypertension	206 (51.2%)	76 (49.4%)	130 (52.4%)	0.608
Depression	28 (7.0%)	12 (7.8%)	16 (6.5%)	0.688
Lung cancer	3 (0.8%)	0 (0%)	3 (1.2%)	0.288
Other cancer	48 (11.9%)	25 (16.2%)	23 (9.3%)	**0.041**
Arterial fibrillation/flutter	26 (6.5%)	10 (6.5%)	16 (6.5%)	1.000
Osteoporosis	5 (1.2%)	4 (2.6%)	1 (0.4%)	0.073
Venous thromboembolism	20 (5.0%)	11 (7.1%)	9 (3.6%)	0.156
Thyroid diseases	77 (19.2%)	46 (29.9%)	31 (12.5%)	** < 0.001**
Sleep apnoea	53 (13.2%)	9 (5.8%)	44 (17.7%)	** < 0.001**
Heart failure	3 (0.7%)	2 (1.3%)	1 (0.4%)	0.561
Kidney disease	22 (5.5%)	7 (4.5%)	15 (6%)	0.654
Diabetes	77 (19.2%)	25 (16.2%)	52 (21%)	0.297
Stroke	18 (4.5%)	2 (1.3%)	16 (6.5%)	**0.014**
Gastro-oesophageal reflux disease	196 (48.8%)	79 (51.3%)	117 (47.2%)	0.473

Significant values are in [bold].

The table contains a comparative analysis of the prevalence of comorbidities in male and female patients within the study population. Data were analysed with Fisher’s exact test; p < 0.05 was considered statistically significant.

### Imaging

CT was assessed visually by experienced thoracic radiologists as part of the radiological findings and during MDD. Reticular opacities were described more frequently in male patients (76.2 vs. 64.9%; p = 0.017), while there were no differences between the sexes with regard to ground-glass opacities or honeycombing. In the evaluation of the radiological patterns, a UIP pattern (either probable or indeterminate) was described more frequently in men (33.9% vs. 16.9%; p < 0.001). Table 3 shows radiological findings and results of lung density histogram analyses.

**Table 3:** Radiological findings and automatically quantified YACTA lung density histogram analyses.

	All N = 402	Females N = 154	Males N = 248	p
Visual assessment				
Ground-glass opacities	146 (58.9%)	98(63.6%)	146 (58.9%)	0.347
Consolidations	94 (40%)	40 (26%)	54 (21.8%)	0.335
Honeycombing	22 (5.5%)	5 (3.2%)	17 (6.9%)	0.608
Reticular opacities	289 (71.9%)	100 (64.9%)	189 (76.2%)	**0.017**
NSIP pattern	206 (51.2%)	85 (55.2%)	121 (48.8%)	0.220
Probable or indeterminate UIP	110 (27.4%)	26 (16.9%)	84 (33.9%)	** < 0.001**
Quantitative analysis*				
Lung volume	4619.0 ± 1307.4	3928,8 ± 979.1	5042.5 ± 1304.8	** < 0.001**
80th percentile	− 664.7 ± 91,6	− 669.1 ± 93.3	− 661.9 ± 90.6	0.481
40th percentile	− 843.5 ± 43.1	− 838.1 ± 43.9	− 846.7 ± 42.3	0.069
EI (%)	0.9 ± 1.9	2.0 ± 1.9	0.9 ± 1.8	0.420

Significant values are in [bold].

The table contains a comparative analysis of the imaging results in male and female patients in the study population. Fisher’s exact test for categorical variables or a two-tailed t test for independent samples in the case of continuous variables; p < 0.05 was considered statistically significant. *Available for N = 304.

### Final diagnoses

**Fig. 1:**
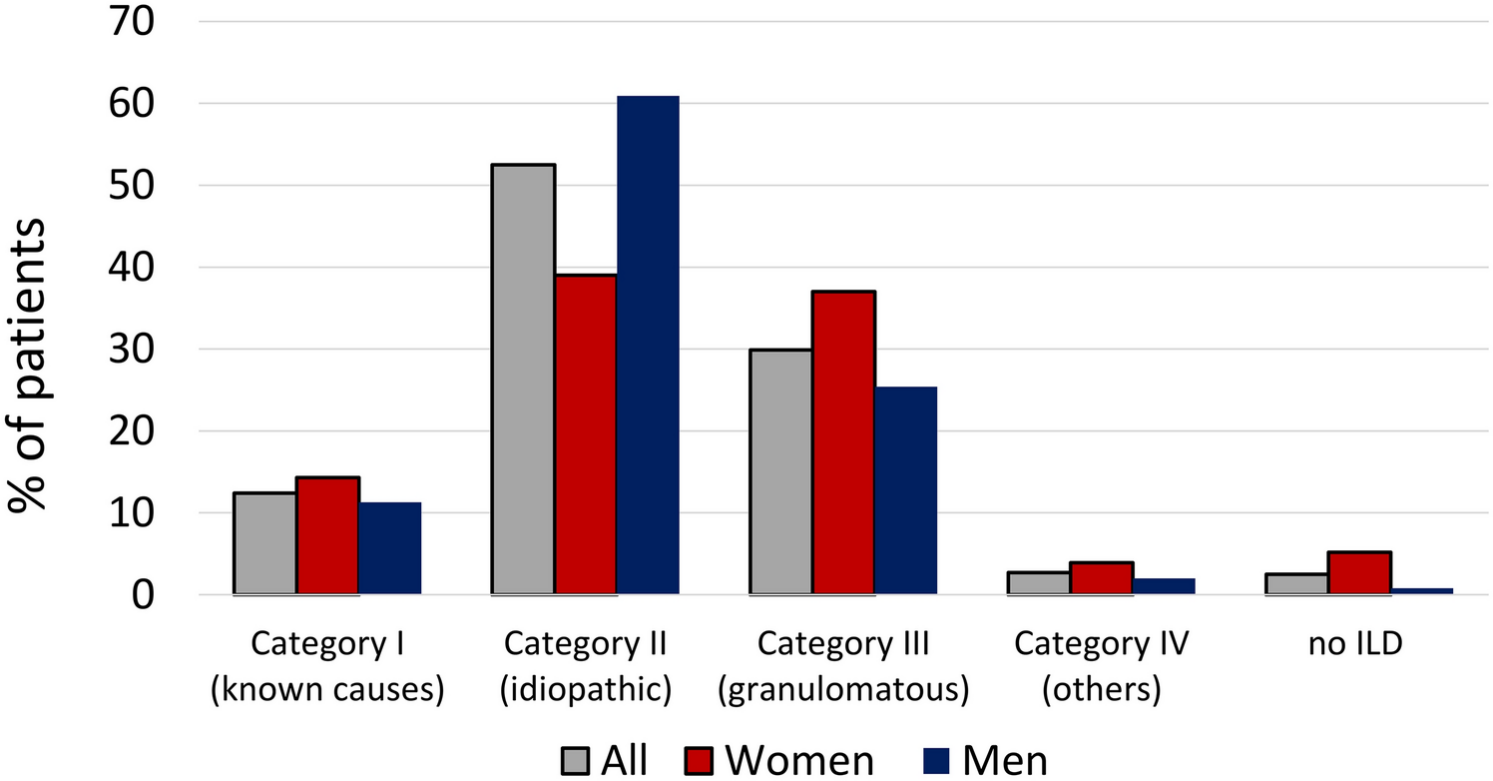
The classification of ILDs according to the final ILD board decision by sexCategory I: ILDs associated with known causes, Category II: idiopathic forms, Category III: granulomatous ILDs, Category IV: others. The figure shows the number of patients in the individual categories as a percentage.

Including the diagnosis of unclassifiable ILD, which was assigned to category II, all but 11 cases that were ultimately not diagnosed with ILD after final assessment, could be assigned to the 4 categories. These were interstitial lung disease with known causes in 12.4% of cases, idiopathic interstitial lung disease in 52.2% of cases, granulomatous interstitial lung disease in 29.9% of cases and others in 2.7% of cases. There were relevant sex-specific differences with regard to ILD categories. The most common diagnostic category, category II, was significantly more common in male patients (60.9% vs. 39%; p < 0.001). The most common diagnosis in this category was IPF, which was diagnosed in 21.8% of men and 7.1% of women (p < 0.001). The second most common diagnosis in this category unclassifiable ILD was also more common in men and was diagnosed in 21.8% of men and 6.5% of women (p < 0.001). In addition, 10.1% of men and 12.3% of women were diagnosed with NSIP, with no differences between the sexes. In contrast, the second most common category, category III, was diagnosed significantly more frequently in women (37% vs. 25.4%; p = 0.007). In terms of the most common diagnoses in this category, HP was more frequent in women (34.4% vs. 21.8%; P = 0.007). There was no sex difference for sarcoidosis, which was diagnosed in 3.2% of men and 1.9% of women. In the third most common diagnostic category, Category I (known causes), the sex prevalences were balanced and there were no sex differences for the individual entities, CTD RA-ILD or drug-induced ILD. Of the category IV diagnoses, lympangioleyomyomatosis was the most common diagnosis, diagnosed in women. Figure 1 provides an overview of the diagnosis categories; an overview of the categories with the most common individual entities is shown in Table 4.

**Table 4:** Final diagnoses, by gender.

	All N = 402	Females N = 154	Males N = 248	p
Category I (known causes)	50 (12.4%)	22 (14.3%)	28 (11.3%)	0.437
RA-ILD	8 (2%)	4 (2.6%)	4 (1.6%)	0.489
CTD-ILD	17 (4.2%)	7 (4.5%)	10 (4%)	0.804
Drug induced	13 (3.2%)	6 (3.9%)	7 (2.8%)	0.572
Category II (idiopathic)	211 (52.5%)	60 (39%)	151 (60.9%)	**< 0.001**
IPF	67 (16.7%)	11 (7.1%)	56 (22.6%)	**< 0.001**
Unclassifiable ILD	63 (15.7%)	19 (12.3%)	44 (17.7%)	0.094
NSIP	44 (10.9%)	19 (12.3%)	25 (10.1%)	0.513
COP	5 (1.2%)	3 (1.9%)	2 (0.8%)	0.376
Category III (granulomatous)	120 (29.9%)	58 (37%)	63 (25.4%)	**0.009**
HP	107 (26.6%)	53 (34.4%)	54(21.8%)	**0.007**
Sarcoidosis	11 (2,7%)	3 (1.9%)	8 (3.2%)	0.543
Category IV (others)	11 (2.7%)	6 (3.9%)	5 (2%)	0.347
LAM	4 (1%)	4 (2.6%)	0 (0%)	**0.021**
No ILD	10 (2.5%)	8 (5.2%)	2 (0.8%)	**0.008**

This table categorizes the most frequent final diagnoses assigned by the multidisciplinary discussion (MDD) team for both male and female patients. Diagnoses are grouped by sex. Data were analysed with Fisher’s exact test; p < 0.05 was considered statistically significant.

RA-ILD Rheumatoid Arthritis-associated Interstitial Lung Disease, CTD-ILD Connective Tissue Disease-associated Interstitial Lung Disease, IPF Idiopathic Pulmonary Fibrosis, NSIP Non-Specific Interstitial Pneumonia, COP Cryptogenic Organizing Pneumonia, LAM Lymphangioleiomyomatosis. Significant values are in [bold]

## Discussion

The objective of the present study was to investigate sex differences in a group of patients with interstitial lung disease with the indication for an extended work-up, including histological confirmation, at a tertiary centre for ILDs with further expert status in interventional endoscopy and thoracic surgery. By focusing on this certain subgroup ILDs where further investigation is indicated, typically a minority in most centres, we aim to explore potential sex-specific differences that could provide valuable insights into the management and diagnostic approach for these patients, particularly those who may not be suitable candidates for invasive diagnostic procedures. Our findings highlight significant sex-based disparities in various aspects of the disease, including risk factors, comorbidities, radiological patterns, and final diagnoses. Notably, idiopathic pulmonary fibrosis (IPF) emerged as the most prevalent diagnosis among men, while hypersensitivity pneumonitis was more frequently diagnosed in women. Additionally, men were more likely to be diagnosed with unclassifiable interstitial lung disease compared to women.

With regard to risk factors, men were more frequently exposed to occupational exposure, while women reported higher exposure to household antigens. Smoking behavior was similar across genders, and CT analyses corroborated the medical history data, showing no differences in the emphysema index between men and women. Comorbidity patterns also differed, with men more likely to have cardiovascular disease such as coronary heart disease and stroke, as well as sleep apnoea. In IPF, the prevalence of coronary heart disease (CHD) was generally high (60%), potentially being a consequence of smoking, which is associated with poorer survival; but an unrecognised comorbid condition in 20% of cases^23^. In our cohort, the prevalence of CHD and IPF was higher in men compared to women, with no differences in smoking behavior between the sexes. We found a high burden of previous malignancies, with 3 cases of lung cancer diagnosed and a total of 48 patients having a history of another malignancy. With 16.2% of women having a previous extrathoracic malignancy, it is also striking that women in this cohort were particularly affected by malignancy. Women were also more likely to have thyroid disease. We observed a significant burden of previous malignancies, including 3 cases of lung cancer and a total of 48 patients with a history of other malignancies. Comorbidities in interstitial lung disease (ILD) may reflect shared risk factors such as smoking but can also represent manifestations of systemic diseases like sarcoidosis or rheumatoid arthritis-related ILD. Furthermore, systemic inflammation is an important factor linking various comorbidities with ILD^24^. In our study, the higher prevalence of thyroid disease in women may be related to the generally increased risk of autoimmune disorders in females^25^. The notable rate of malignancies in women is surprising and deserves further investigation. One potential explanation could be systemic inflammation, which is known to be involved in tumorigenesis^26^.

We observed no sex differences in the severity of lung disease among our patients, as indicated by static and dynamic lung volumes, diffusion capacity, and histogram analyses of lung density. The qualitative differences became evident only through the analysis by an experienced thoracic radiologist, with reticular opacities and UIP features being more prevalent in men. These radiological indicators of IPF corresponded with the final diagnoses. There were no gender-specific differences in the systematically conducted autoantibody screenings. It is important to note that patients with ILD associated with RA, CTD, or SSc typically do not require histological confirmation of the diagnosis. Therefore, those with autoimmune diseases might represent a specific subset of cases. It is also noteworthy that although a diagnosis could be made in nearly all cases after the second multidisciplinary discussion (MDD), a diagnosis of ‘unclassifiable ILD’ was notably more frequent in men. It is important to critically assess that our MDD process is not gender-blind, which could potentially introduce biases in the final diagnoses. Research by Assayag et al. indicates that patient sex independently influences the diagnosis of IPF. Their study found that pulmonologists were more likely to diagnose idiopathic pulmonary fibrosis in male patients, even after adjusting for factors such as age, smoking history, exposures, and autoantibodies. This suggests that clinicians may place significant emphasis on male sex when assessing the pre-test diagnostic probability of this disease^27^. This could ultimately lead to underdiagnosis of IPF in women and overdiagnosis in men. The relatively low proportion of women in IPF cohorts has resulted in a significant gender bias in the existing literature on IPF, with the majority of studies focusing on male subjects. Consequently, there is a dearth of research that explicitly examines the characteristics and outcomes of women with IPF. Data from the French ILD cohort "COhorte Fibrose (COFI)" show that IPF looks different in women than in men, the majority of women in the COFI cohort were neversmokers in 65% of cases, compared to a rate of 21% in men^28^. They were also less likely to present with honeycombing or concomitant emphysema. These findings are supported by data from the French Rare Disease Cohort—Interstitial Lung Disease (RaDiCo-ILD), where comorbid emphysema, and honeycombing on HRCT are more common characteristics of males than females with IPF^29^. Although histological characteristics of IPF may vary between sexes, we assume that women with IPF, potentially underdiagnosed due to distinct clinical phenotypes and possible investigator bias, would particularly benefit from invasive diagnostic methods for accurate diagnosis. The higher prevalence of HP in women in our study was surprising. Although there are data from some registries, such as the Danish one, where men have a slightly higher prevalence (57%) than women^30^, it is generally assumed that the gender-specific prevalence of this entity is relatively balanced^31^. However, antigen-indeterminate cases are reported more often in older women with lower lung function, less alveolar lymphocytosis and greater fibrosis on imaging^32^. The fact that histological clarification may be indicated in these cases in particular may explain the higher prevalence of the disease in our collective.

### Limitations

This study has several limitations. First, the sex data are based on patient self-report, which may introduce potential biases. While all patients were examined according to internal center standards, the quality of the data cannot be compared with that of a controlled study. CT scans were interpreted by experienced radiologists, who, like other members of the MDD team, may have been influenced by potential bias. Additionally, diagnoses of comorbidities were primarily based on pre-existing medical records, which were not consistently reviewed at the center. The monocentric nature of the study and the absence of a control group or matched cohorts limit the generalizability of the findings. Furthermore, 62% of the patients were male, and patients deemed ineligible for transbronchial cryobiopsy due to comorbidities, disease severity, or refusal were not included. The monocentric nature of the study and the absence of a control group or matched cohorts limit the generalizability of the findings. Furthermore, 62% of the patients were male, and patients deemed ineligible for transbronchial cryobiopsy (TCB) due to comorbidities, disease severity, or refusal were not included. Finally, prospective, multicentric studies are needed to validate these findings and explore the role of sex-specific differences in ILD diagnosis and outcomes.

## Conclusion

This work offers a preliminary overview of sex differences in a well- characterised real-world cohort investigated at a single centre using transbronchial cryobiopsy. The high prevalence of IPF among men and, conversely, the more frequent diagnosis of hypersensitivity pneumonitis (among women had therapeutic consequences, making invasive diagnostic work-up helpful for these patients. Overall, it is possible that men and women may require different therapeutic approaches. However, it is important to emphasize that treatment decisions must be made based on the individual diagnosis, considering the unique characteristics of each patient and their response to therapy. The study emphasizes the importance of understanding these disparities to improve patient outcomes and guide future research directions in the field of interstitial lung disease.

## Data Availability

The full data set supporting the conclusions of this article is available upon request from Franziska C. Trudzinski.

## Abbreviations

ATS: American Thoracic Society
BAL: Bronchoalveolar lavage
BMI: Body Mass Index
CHD: Coronary heart disease
COFI: Cohorte Fibrose
CTD: Connective tissue diseases
DLCO: Diffusion capacity for carbon monoxide
ERS: European Respiratory Society
FEV1: Forced expiratory volume in the first second
FVC: Forced vital capacity
GLI: Global Lung Function Initiative
HP: Hypersensitivity Pneumonitis
HU: Hounsfield-Unit
IIPs: Idiopathic Interstitial pneumonias
ILD: Interstitial lung disease
iNSIP: Idiopathic non-specific interstitial pneumonitis
IPF: Idiopathic pulmonary fibrosis
LAM: Lymphangioleiomyomatosis
MDCT: Multidetector computed tomography
MDD: Multidisciplinary discussion
RA: Rheumatoid arthritis
RaDiCo-ILD: Rare Disease Cohor-Interstitial Lung Disease
SD: Standard deviations
SLB: Surgical lung biopsy
SSc: Systemic sclerosis
TCB: Transbronchial cryobiopsy
UIP: Usual interstitial pneumonia
VATS: Video-assisted thoracoscopic surgery
